# Alginate-Based Hydrogels Enriched with Lavender Essential Oil: Evaluation of Physicochemical Properties, Antimicrobial Activity, and In Vivo Biocompatibility

**DOI:** 10.3390/pharmaceutics15112608

**Published:** 2023-11-09

**Authors:** Alina Gabriela Rusu, Loredana Elena Niță, Irina Roșca, Alexandra Croitoriu, Alina Ghilan, Liliana Mititelu-Tarțău, Aurica Valentin Grigoraș, Bianca-Elena-Beatrice Crețu, Aurica P. Chiriac

**Affiliations:** 1 Natural Polymers, Bioactive and Biocompatible Materials Department, “Petru Poni” Institute of Macromolecular Chemistry, 41-A Grigore Ghica Voda Alley, 700487 Iasi, Romania; lnazare@icmpp.ro (L.E.N.); croitoriu.alexandra@icmpp.ro (A.C.); diaconu.alina@icmpp.ro (A.G.); cretu.bianca@icmpp.ro (B.-E.-B.C.); achiriac@icmpp.ro (A.P.C.); 2 Center of Advanced Research in Bionanoconjugates and Biopolymers, “Petru Poni” Institute of Macromolecular Chemistry, 41-A Grigore Ghica Voda Alley, 700487 Iasi, Romania; rosca.irina@icmpp.ro; 3 Department of Pharmacology, Clinical Pharmacology and Algesiology, “Grigore T. Popa” University of Medicine and Pharmacy, Universitǎţii Street 16, 700115 Iasi, Romania; lylytartau@yahoo.com; 4 Stejarul Research Centre for Biological Sciences, National Institute of Research and Development for Biological Sciences, Alexandru cel Bun Street, 6, 610004 Piatra Neamț, Romania; valygrigoras@yahoo.com

**Keywords:** hydrogels, antimicrobial activity, lavender essential oil, alginate

## Abstract

Owing to its antibacterial, anti-inflammatory, and antioxidant activities, in the last few years, lavender essential oil (LVO) has been used in medical applications as a promising approach for treating infected wounds. However, the practical applicability of LVO is limited by its high volatility and storage stability. This study aimed to develop a novel hybrid hydrogel by combining phytic acid (PA)-crosslinked sodium alginate (SA) and poly(itaconic anhydride-co-3,9-divinyl-2,4,8,10-tetraoxaspiro[5.5] undecane (PITAU) and evaluate its potential effectiveness as an antibacterial wound dressing after incorporating LVO. The influence of the mass ratio between SA and PITAU on the properties and stability of hydrogels was investigated. After LVO loading, the effect of oil addition to hydrogels on their functional properties and associated structural changes was studied. FTIR analysis revealed that hydrogen bonding is the primary interaction mechanism between components in the hybrid hydrogels. The morphology was analyzed using SEM, evidencing a porosity dependent on the ratio between SA and PITAU, while LVO droplets were well dispersed in the polymer blend. The release of LVO from the hydrogels was determined using UV-VIS spectroscopy, indicating a sustained release over time, independent of the LVO concentration. In addition, the hybrid hydrogels were tested for their antioxidant properties and antimicrobial activity against Gram-positive and Gram-negative bacteria. Very good antimicrobial activity was obtained in the case of sample SA_PITAU3+LVO10% against *S. aureus* and *C. albicans*. Moreover, in vivo tests showed an increased antioxidant effect of the SA_PITAU3+LVO10% hydrogel compared to the oil-free scaffold that may aid in accelerating the healing process of wounds.

## 1. Introduction

In order to ease problems caused by antibiotic resistance, the use of bioactive compounds extracted from medicinal plants has emerged as a highly noteworthy area of research in the field of medical and pharmaceutical applications [1,2,3]. Due to their high bactericidal action against a variety of bacterial pathogens, studies have demonstrated that natural compounds like plant essential oils [2,4,5] hold enormous promise as antibacterial agents. The fact that essential oils and their components are quickly digested, do not build up in the body, and are quickly excreted after application to the skin strongly suggests that they can be used as effective antimicrobial agents [6]. The incorporation in carriers or scaffolds of such natural compounds derived from medicinal plants has gained a lot of interest and demand, especially in replacing antimicrobial drugs due to their minimal side effects and relative abundance [7,8].

After extraction of those active substances, their stability over time is an important aspect that must be taken care of, as it can be significantly impacted by environmental factors like light, heat, and moisture.

In order to maintain all the distinctive properties of these essential oils until they reach their desired physiological targets, several methods have been introduced [9,10,11] and encapsulation in different materials has emerged as the most effective [12,13].

Lavender essential oil (LVO) is extracted from many species of lavender plants, such as *Lavandula latifolia*, *Lavandula stoechas*, and *Lavandula angustifolia*, which exhibit excellent antimicrobial, antifungal, and antioxidant properties [14,15,16]. The antibacterial activity of LVO has been investigated on several types of microorganisms, with notable effectiveness observed against *Staphylococcus aureus*, *Micrococcus ascoformans*, *Proteus vulgaris*, *Escherichia coli*, *Pseudomonas aeruginosa*, and *Candida albicans* [17,18]. Moreover, LVO effectively prevents the growth of microorganisms that cause infections on the skin and is often used for the treatment of surface infections as a topical or prophylactic application [19,20]. According to some published studies, linalool and linalyl acetate are the major constituents of LVO and together with other minor components are responsible for its antimicrobial efficiency. Moreover, linalool and linalyl acetate also have an anti-inflammatory effect [21,22]. The incorporation of LVO in hydrogels improves the oil stability during storage [23], delays its release under real-time dynamic conditions, and imprints good antibacterial properties against both Gram-negative and Gram-positive bacteria, making it a key factor in accelerating the stages of wound healing [24].

Due to their structural similarities to the extracellular matrix (ECM) found in the native skin, polymeric scaffolds like hydrogels, particularly those originating from natural polymers, have become one of the most promising therapeutic options for managing wound healing [25,26].

Loading the hydrophobic essential oil into the hydrophilic structure of a hydrogel requires dispersing substances such as tweens, spans, and cyclodextrins, which are usually utilized. Moreover, solid particles like halloysite nanotubes [27] or hydrophobic polymers (polycaprolactone) dissolved in the organic phase of an oil in water (O/W) emulsion [28] were utilized to stabilize the essential oils droplets into the hydrogel matrix.

Our group successfully developed a copolymer through radical copolymerization of itaconic anhydride and 3,9-divinyl-2,4,8,10-tetraoxaspiro[5.5] undecane (poly(itaconic anhydride-co-3,9-divinyl-2,4,8,10-tetraoxaspiro[5.5] undecane -PITAU) with unique properties including binding ability, thermal stability, and sensitivity to changes in pH and temperature [29]. Also, it has been shown that PITAU has the unique capacity to establish specific functional pathways and further link biological molecules via the itaconic anhydride moiety (riboflavin [30,31]) exhibiting biologically desirable characteristics such as biodegradability and biocompatibility. These superior properties make this polymer highly promising for pharmaceutical delivery and supporting bioactive compounds in various biomedical applications.

Alginate (SA), a polysaccharide extracted from brown seaweed, is nowadays utilized increasingly as a carrier to transport active compounds and in the production of hydrogels due to its biodegradability, nontoxicity, low cost, and ease of gelation [32,33]. Also, SA has film-forming properties and produces impermeable films to oils and lipids [34]. Moreover, they are effective barriers against oxygen [34], and can also slow down lipid oxidation [35]. However, the films have reduced moisture retention and are water-soluble [36], requiring crosslinking and/or combination with a synthetic polymer to improve their properties. Also, a unique property of SA is its ability to form an insoluble gel through ionotropic gelation by multivalent cations such as Ca^2+,^ Sr^2+^, Ba^2+^, Zn^2+^, Mn^2+^, Fe^2+^, Cr^3+^, and Fe^3+^ that crosslink carboxylate groups in the uronate blocks [37]. Thus, selecting the appropriate crosslinking agent is crucial for scaffold formation and high performance in specific environments. Phytic acid (myo-inositol-1,2,3,4,5,6-hexakisphosphate, PA) is a natural antioxidant found in abundance in plant seeds [38]. It has many hydroxyl-bearing phosphoric groups and it is known for its great antioxidant and chelating properties already demonstrating the ability to form functional materials that hold great potential for biomedicine [38]. Through the combination of PA with natural polymers like carboxymethyl cellulose [39], SA [40], and carboxymethyl chitosan [41], naturally crosslinked scaffolds were obtained. This process can occur through the bonding of PA anions to cationic groups of the polymer or by forming hydrogen bonds from the hydroxyl groups within the compound’s structure [42]. Previously, in our group, hydrogels based on (i) SA crosslinked with PA [40] and (ii) hybrid gels composed from SA and PITAU [43] were prepared and characterized.

Taking into account the capacity of PITAU to form a network and incorporate bioactive compounds, as well as the hybrid hydrogel systems above mentioned, in the present study, we developed this direction by investigating a complex hydrogel made by interpenetrating PITAU copolymer loaded with LVO in the preformed network based on SA biopolymer crosslinked with PA. The new platform was explored for synergistic properties regarding the application as antibacterial wound dressings.

The purpose was to obtain a hybrid scaffold loaded with LVO that promotes a controlled release of the oil in the first hours to decrease the bacterial infection and after, through a sustained stage, slowly reduces the oxidative stress in the cells, leading to rapid wound healing. The physicochemical properties of the produced hydrogels were investigated, and the optimum scaffold was embedded with LVO to induce antibacterial and antioxidant properties.

Together with their antimicrobial activity, other properties of the composite hydrogels have been studied such as their swelling capacity, antioxidant activity, and release rate of LVO.

## 2. Materials and Methods

### 2.1. Chemicals

All chemicals obtained from commercial suppliers were of analytical purity and were used without further purification: phytic acid (PA, 50 wt% in water) purchased from Sigma-Aldrich (Darmstadt, Germany), alginic acid sodium salt (SA) from brown algae supplied by Across Organics (Geel, Belgium), 3,9-divinyl-2,4,8,10-tetraoxaspiro[5.5] undecane (U) (purity 98%, Sigma-Aldrich, Hamburg, Germany), itaconic anhydride (ITA) (purity 98%, Aldrich, Darmstadt, Germany), 2,2′-Azobis (2-methyl propionitrile) (AIBN) (purity 98%, Sigma-Aldrich, Darmstadt, Germany), lauryl sulfate sodium salt (SLS), and 2,2-diphenyl-1-picrylhydrazyl (DPPH) (Sigma-Aldrich, Darmstadt, Germany).

The molecular weight of the SA used in this study was determined with the static light scattering technique using the Zetasizer Nano ZS equipment and was found to be around 90 kDa, which is in agreement with other investigation in the field [44]. Its chemical composition, as determined using ^1^H NMR spectroscopy, showed a ratio between mannuronic (M) and guluronic (G) acids of 1.22 M/G [45]. The organic solvents used in the synthesis of PITAU were 1,4 dioxane (D) (purity ≥ 99.0%) and diethyl ether (for precipitation), purchased from Sigma-Aldrich (Darmstadt, Germany). Ultra Clear TWF UV System was used to purify the water utilized in the experiments.

### 2.2. Hybrid Hydrogels Preparation

Several hybrid hydrogels based on PITAU and SA and crosslinked with PA were prepared and their codification and chemical composition is presented in Table 1. Before mixing with the synthetic copolymer, the SA solution (3%) was crosslinked with a PA solution (10%). According to previous studies [40], the ratio of 6:1 (wt/wt) between SA and PA was optimal for preparing stable PA-crosslinked SA hydrogels; therefore, for all prepared hydrogels, the SA: PA mass ratio was kept constant. Shortly after, specific amounts of crosslinked SA solution were mixed with PITAU copolymer (in dioxane solution) to achieve gravimetric SA: PITAU ratios of 0.3:1; 0.45:1, and 0.6:1. The gels formed within 20 min after mixing were left to mature for 24 h. Then, the gels were freeze-dried to remove the solvents and to be used in further investigations.

### 2.3. Extraction and Analysis of the Essential Oil from Lavender Flowers

The essential oil was extracted using *Lavanda angustifolia Mill*., Codreanca variety; the raw material was harvested in mid-July 2022 from the experimental fields of the Agricultural Research and Development Station, Secuieni, Neamt County, Romania. The lavender culture is an ecological, non-irrigated one and is in its fourth year of development. The harvesting was carried out in ideal conditions: at noon and under a clear sky (temperature in the field of 32–40 °C). After drying for 14 days at room temperature and controlled humidity, the plant material was processed following the European Pharmacopoeia [46]: the dried flowers were manually separated from the flowering stem and grinded for 1 min. Immediately after, the essential oil was extracted by hydrodistillation, using a Neo-Clevenger type apparatus. The content in the plant sample was determined according to the European Pharmacopoeia: 20 g of dry and crushed plant material was transferred in a 1000 mL round-bottom flask; 500 mL of distilled water and 0.5 mL of hexane were added, and the mixture was hydrodistillated for 2 h. At the end, the oil sample was collected and stored in the refrigerator until the time of analysis. The average essential oil content was 3.20 ± 0.22 mL/100 g of dry plant material.

The essential oil obtained was subjected to GC-MS analysis (gas chromatography) in order to evaluate its composition. The analysis of the sample was performed using an Agilent 7890A gas chromatograph, equipped with an Agilent 5975C mass spectrometer and a DB-5MS capillary column (30 m × 0.25 mm internal diameter, 0.25 µm film thickness). The carrier gas was helium with a flow rate of 1 mL/min. The injector (split ratio 100:1) and detector temperatures were maintained at 250 °C. The volume injected was 0.25 µL. The oven temperature was stable at 40 °C for 3 min, then raised with 10 °C/min up to 280 °C. The final temperature was kept constant for 3 min. The evaluation of the results was performed using ChemStation Software (version E.02.02) and Wiley Mass Spectral Library.

### 2.4. Bioactive Compound Preparation

LVO-loaded SA/PA/PITAU hydrogels were prepared using the same protocol established for hydrogels preparation, described in the section above. The compound ratios used in the bioactive hydrogel development are presented in Table 2.

Thus, LVO was mixed with PITAU solution solutions at concentrations of 5%, 10%, and 15% dripped in the preformed network of SA crosslinked with PA and stirred for at least 15 min to obtain a homogenous mixture. The ratio of 0.6:1 between SA and PITAU was chosen for LVO embedding, as this ratio was the optimal one that allowed for a better interaction between the hybrid polymer network and the essential oil. The encapsulation efficiency *w/w*% (*EE*%) and loading capacity *w/w*% (*LC*%) of LVO were also calculated using equations presented in a section below and included in Table 2.

### 2.5. Structural Characterization

The FTIR spectra of both precursors and hydrogels were obtained using a Vertex Bruker spectrophotometer (Ettlingen, Germany) in transmittance mode. The spectra were recorded at room temperature in a wave number range of 4000–400 cm^−1^ with 64 scans and a resolution accuracy of 4 cm^−1^.

### 2.6. Morphological Analysis

To observe the morphology of the freeze-dried hydrogels, a scanning electron microscope (SEM Quanta 200, FEI Company, Hillsboro, OR, USA) was used. The instrument was operated in low-vacuum mode with secondary electrons at 20 kV and without any coating. The samples were first fixed onto aluminum stubs with double-adhesive carbon tape before analysis.

### 2.7. Swelling Studies

Swelling measurements of hydrogels were performed by using the teabag method, in phosphate buffer solution at pH 7.4 and 37 °C [47]. Briefly, 20 mg of lyophilized hydrogel was weighed into an empty teabag and then immersed in media. At specific time intervals, the samples were taken out from the buffer solution, and the excess was blotted with filter paper and then weighed [48]. The weight of the swollen gel was obtained by subtracting the weight of the wet teabag from the total weight. Swelling studies of all hydrogels were carried out three times and the mean values were reported. The degree of swelling (SD) was calculated using the following Equation (1):

(1) $$SD\;\left(\%\right)=\left(\frac{W_{3}-W_{2}-W_{1}}{W_{2}}\right)\times 100$$

where *W*_1_, *W*_2_, and *W*_3_ are the weight of the teabag, the weight of the dried hydrogel, and the weight of the swelled hydrogel, respectively.

For the pH sensitivity evaluation, the swelling behavior of the prepared hydrogels in buffer solutions with different pHs (5.4, 6.5, and 7.4) was measured after reaching the equilibrium state, also according to the teabag method.

### 2.8. LVO Loading and Release Assays

#### 2.8.1. LVO Loading

The LVO content loaded in the hydrogels was determined using UV–VIS spectrophotometry [23,49,50]. Each hydrogel (20 mg) was dissolved in 4 mL SLS solution (6 *w/v*%) under magnetic stirring, and the content of LVO was determined by using a UV-VIS spectrophotometer (Jenway 6305 UV–VIS Spectrophotometer, Stone, Staffordshire, UK) at a wavelength of 276 nm. The amount of LVO was calculated by using a previously constructed calibration curve of free LVO in a 6% SLS solution, which was standardized with a limit of detection of 0.064 mg/ mL and a limit of quantification of 0.194 mg/mL. Triplicate measurements were performed for each sample. The correlation coefficient, regressive equation, and linear range are detailed in the supplementary material, Figure S1. The *EE%* and *LC%* of LVO were calculated according to Equation (2) and Equation (3), respectively, as follows:

(2) $$EE\%=\frac{Total\;amount\;of\;loaded\;LVO(\%\;in\;hydrogel)}{Initial\;amount\;of\;LVO(\%\;in\;in\;itial\;solution)}\times 100$$

(3) $$LC\%=\frac{Total\;amount\;of\;loaded\;LVO(amount\;in\;oil)}{Weight\;of\;hydrogel\;after\;loading}\times 100$$

#### 2.8.2. Release Study

To study the release percentage of LVO from hydrogels, the samples (50 mg) were introduced in a dialysis bag and immersed in 20 mL buffer solution (pH 7.4) containing 1% SLS (*w/v*%). All experiments were performed at 37 °C, under gentle shaking. At certain time intervals, from each incubation medium, 2 mL was withdrawn and replaced with the same volume of freshly prepared buffer with SLS 1%, standardized with a limit of detection of 0.052 mg/mL and a limit of quantification of 0.156 mg/mL. The correlation coefficient, regressive equation, and linear range are also listed in the supplementary material, Figure S2. The amount of released lavender was then quantified using UV–VIS spectrophotometry (Jenway 6305 UV–VIS Spectrophotometer, Stone, Staffordshire, UK) at λ = 276 nm [49].

### 2.9. Antioxidant Capacity of the Hybrid Hydrogels

The antioxidant activity of the LVO-loaded/oil-free hydrogels was determined by measuring the 2,2-diphenyl-1-picrylhydrazyl (DPPH) scavenging capacity. The DPPH radical scavenging assay was performed according to the Blois method [51]. In brief, 20 mg from each hydrogel loaded with LVO was swelled in 3 mL ethanol and centrifuged to remove the insoluble hydrogel fraction. Separately, a 0.5 mM DPPH stock solution in ethanol was prepared and then 0.3 mL of this solution was mixed with 3 mL of the supernatant hydrogel solution. The control solution was prepared by mixing ethanol (3 mL) and DPPH stock solution (0.3 mL). Finally, after 30 min of incubation in the darkness, the absorbance was measured at 517 nm (Jenway 6305 UV–VIS Spectrophotometer, Stone, Staffordshire, UK). All experiments were performed in triplicate and the results were reported as mean ± SD of percent radical scavenging activity. The percentage of DPPH radical scavenging was calculated by the following equation:

(4) $$\%DPPH\;radical\;scavenging\;activity=\frac{\left(A_{c}-A_{s}\right)}{A_{c}}\times 100$$

where *A_c_* is the absorbance of the control DPPH solution and *A_s_* is the absorbance of the DPPH solution containing the analyzed samples.

### 2.10. Antimicrobial Activity

By using the disk diffusion test [52,53] against three distinct reference strains of *Staphylococcus aureus* ATCC25923 (*S. aureus*), *Escherichia coli* ATCC25922 (*E. coli*), and *Candida albicans* ATCC90028 (*C. albicans*), the antimicrobial activity screening of the samples was assessed. All microorganisms were kept in 20% glycerol at −80 °C. The yeast strain was refreshed on Sabouraud dextrose agar (SDA) at 37 °C, while the bacterial strains were refreshed on nutrient agar (NA) at that same temperature. These cultures were used to create microbial suspensions in a sterile solution, resulting in turbidity optically similar to that of 0.5 McFarland standards. On NA/SDA plates, samples of 25 mg and 10 mm were added to 100 μL samples from each inoculum spread on the Petri dishes.

After 24 h of incubation at 37 °C, the growth inhibition was assessed under standard conditions to evaluate the antimicrobial properties. To ensure the accuracy of the results, each test was carried out three times. The samples were examined after incubation using SCAN1200^®^, version 8.6.10.0 (Interscience, Saint-Nom-la-Bretèche, France), and the results were reported as the mean ± standard deviation (SD) using XLSTAT Ecology version 2019.4.1 software [54].

### 2.11. In Vivo Biocompatibility Assay

In the experiment on biocompatibility testing, adult white Wistar rats, male, with uniform sex distribution were used, weighing between 225 g and 250 g and being 6–8 weeks old at the start of the experiments. The animals were purchased from the “Cantacuzino National Medical-Military Institute for Research and Development”, Băneasa Station, Bucharest, Romania through the CEMEX biobase (“Advanced Center for Research and Development in Experimental Medicine”) of the University of Medicine and Pharmacy “Grigore T. Popa” from Iaşi.

The in vivo experiments took place in specially designed enclosures, ensuring the habitat conditions according to standard norms. Rats were housed in individual cages outside of testing periods and provided with food and water ad libitum. A 12 h light/12 h dark cycle was used. The temperature in the laboratory was 23 ± 2 °C, and the relative humidity was 50–60%. The animals were weighed before each administration, the dose being adapted to the weight variation of the animal during the experiment.

At the beginning of the experiment, the animals were anaesthetized with a mixture of ketamine (50 mg/kg) and xylazine (10 mg/kg), shaved in the lateral dorsal region, and an incision was made where sterile cotton pellets, respectively, the hydrogels to be tested, with approximately equal weights (64 mg) were placed subcutaneously. After the end of the experiments, the rats were euthanized under general anesthesia with 3% isoflurane [55,56].

To conduct the in vivo biocompatibility tests, 5 groups each containing 5 rats were used. Two groups (SA_PITAU_3_ and SA_PITAU_3_+LVO10%) were dedicated to the evaluation of newly prepared hydrogels samples, while one group was used for the negative control coded Cn (bi-distilled water), one as the positive control coded Cp (cotton pellet) and another one used implant of pellets impregnated with diclofenac sodium 15 mg/kg body. The weights of the animals in the experimental groups were monitored periodically, to follow the evolution of their weight, during the experiment.

Before the subcutaneous implantation of the tested sample (baseline) and on the 7th day of the experiment, blood samples (2 mL) from the lateral caudal vein [57] were collected and subjected to analysis of hemodynamic, immune, and biochemical profiles.

The following determinations were made: white blood cell percentage (polymorphonuclear neutrophils (PMN), lymphocytes (Ly), eosinophils (E), monocytes (M), and basophils (B)—described in the Supplementary Material, Figures S4–S6), fibrinogen and C-reactive protein, the activity of liver enzymes: aspartate aminotransferase (AST) and alanine aminotransferase (ALT), kidney function: serum urea and creatinine levels, immune parameters: serum complement (C) [58], serum levels of tumor necrosis factor alpha (TNFα) and interleukin 10 (IL-10), and assessment of oxidative stress through measuring superoxide dismutase (SOD) [59] and malondialdehyde (MDA) activity [60].

### 2.12. Statistical Analysis

All the investigations were conducted in triplicate. Results were expressed as the mean ± standard deviation (S.D.) of each evaluated parameter.

For in vivo tests, the data were analyzed statistically using SPSS software (version 17.0) for Windows and the one-factor ANOVA method. Values were expressed as arithmetic mean ± D.S. of the mean for 5 rats per lot. The statistical analysis was completed with the post-hoc Tukey and Newman–Keuls tests, which allowed for multiple comparisons to be made, the separation of the batches into groups of significance, in relation to the intensity of the effect, and the ranking in ascending order of the potency of the tested substances. A *p*-probability of less than 0.05 and 0.01, respectively, was considered significant compared to the control group.

### 2.13. Research Ethics

The in vivo experiments complied with the ethical norms in experimental research, as well as the conditions imposed by the Ethics Commission of the “Grigore T. Popa” University of Medicine and Pharmacy in Iaşi under the European Union norms regarding the work on laboratory animals (“Directive 2010/63/EU of the European Parliament and of the Council of 22 September 2010 on the protection of animals used for scientific purposes”).

## 3. Results and Discussions

LVO is an essential oil with excellent antibacterial, antifungal, and antioxidant properties. Additionally, LVO effectively prevents the growth of microorganisms that cause skin infections and is often used to treat superficial infections in topical or prophylactic form. Incorporation of LVO into hydrogels can improve the stability of LVO during storage, delay its release, and provide good antibacterial properties against Gram-negative and Gram-positive bacteria, making it a key factor in accelerating the stages of wound healing. Due to their structural similarity to the native extracellular matrix (ECM), polymeric scaffolds, such as hydrogels based on natural polymers, have become one of the most promising treatment options to control the wound healing process. Thus, by loading LVO into a hybrid hydrogel made of PITAU copolymer, a compound having the capacity to bind biological molecules via the itaconic anhydride moiety, and SA crosslinked with PA, it is possible to obtain antibacterial hybrid hydrogel for wound treatment.

### 3.1. FTIR Analysis

**Figure 1:**
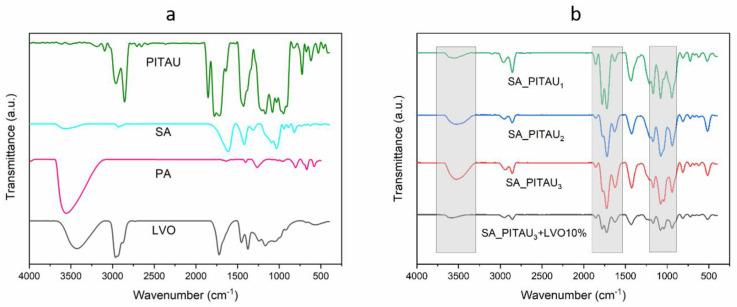
FTIR spectra of (**a**) precursor (SA, PA, and PITAU) and (**b**) hybrid hydrogels.

The FTIR spectra of the gel samples are shown in Figure 1. In all SA/PA/PITAU hydrogels, the characteristic peaks in the domain 3500–3400 cm^−1^ are attributed to –OH stretching vibrations of SA and PA compounds [40]. A couple of bands that appear in the region 2930–2850 cm^−1^ are assigned to stretching vibrations of aliphatic CH and correspond to PITAU [29]. Other characteristic bands of the PITAU copolymer that indicates its presence appeared around 1772 cm^−1^ and 1855 cm^−1^ corresponding to C=O symmetric and asymmetric stretching of the five-member anhydride unit and at 1630 (1624) cm^−1^ from C=C stretching vibration [29]. The absorption bands that appear around 1700 cm^−1^ in all hydrogels spectra correspond to the stretching vibration of the hexatomic ring in PA and are overlapped with bands from the PITAU spectrum mentioned above [40]. The aforementioned peaks have an enhanced intensity as compared to the bands from the PITAU spectrum, which confirms a strong interaction between SA and PA. The presence of a strong band around the 1000–1200 cm^−1^ region may be also attributed to ether C–O–C stretching from the spiroacetal moieties that are overlapped with the C–O–C stretching vibrations of SA saccharide ring and the anti-symmetrical frequencies of the P-O-C groups [29,40]. The band from 673 cm^−1^ corresponding to the bending vibration of P-O bonds, and attested by other authors, is also evidenced [40].

The absorption band of the hydrogels from 1419 cm^−1^, assigned to the symmetric stretching vibration of COO^−^, was shifted to 1433 cm^−1^ for SA_PITAU_1_, respectively, to 1425 cm^−1^ in the case of SA_PITAU_2_ and SA_PITAU_3_. Moreover, as compared to the SA compound spectrum, the band characteristic of OH groups from SA and PA changes the position to lower frequencies in SA/PA/PITAU hydrogels: from 3564 to 3554 cm^−1^ for SA_PITAU_1_ and 3525 cm^−1^ for SA_PITAU_2_ and SA_PITAU_3_. These shifts confirm the formation of hydrogen bonds between SA and the crosslinking agent (PA) within the new hydrogels.

Regarding the addition of LVO, its presence in the new hydrogels network is confirmed by the appearance of new bands at 1338 cm^−1^, 1240 cm^−1^, and 1213 cm^−1^ corresponding to the C–O stretching vibration of ester groups from linalyl acetate and lavandulyl acetate (Figure 1b) [61,62]. Overall, no other major occurrences of new bands or shifting of the old ones were noted in the spectrum of LVO-loaded SA_PITAU_3_ hydrogel. Most of the bands characteristic to the main components of LVO such as linalool, linalyl acetate, lavandulyl acetate, 1,8-cineol, and β-caryophyllene overlap with the bands from the polymeric matrix constituents. These results are consistent with other published work [63]. Based on the above observations, it can be concluded that the hydrogel effectively incorporates LVO.

### 3.2. Morphological Analysis

**Figure 2:**
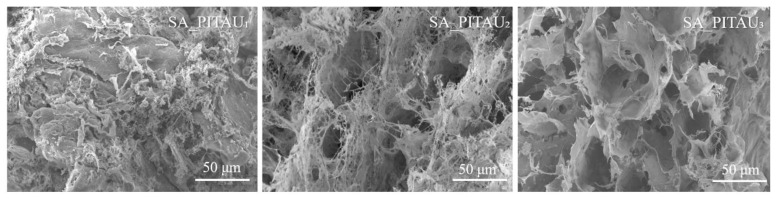
SEM images of hydrogels based on SA, PA, and PITAU.

The architecture of SA/PA/PITAU hydrogels in different ratios was analyzed through the SEM technique and the images are displayed in Figure 2. SEM image analysis can provide a correlation between the bioconjugate matrices’ morphology and the gel’s ability to swell, embed, transport, and release a therapeutic drug or even essential oils.

The freeze-dried hydrogels all have a porous network with different patterns, which is related to the variation in SA and PITAU ratio. When a higher amount of SA was incorporated, the pore diameter became smaller (62.5 ± 0.1 µm for SA_PITAU_2_ and 32.4 ± 0.1 µm for SA_PITAU_3_). This indicates that additional intermolecular crosslinking bridges are developing as a result of the interaction with PA. On the other hand, the network obtained from SA_PITAU_1_ is more compact without visible pores. However, by increasing the SA amount (SA_PITAU_2_ and SA_PITAU_3_), the scaffold network becomes more porous with well-defined and interconnected pores. Thus, a large amount of PITAU causes the appearance of supplementary intermolecular interactions with the already formed SA/PA network. This translates into obtaining a more compact network.

The hydrogel’s pores contributed to the product’s increased ability to swell. The region through which water molecules permeate and interact with the hydrophilic groups of the hydrogel is represented by the hydrogel pores. The domains of PITAU copolymer in the new hydrogels are of great interest for bioactive compound loading like essential oils (LVO), as they can contribute to a controlled release over time in a sustained manner.

### 3.3. Swelling Capacity Evaluation

The capacity of a hydrogel to swell is influenced by various factors, including pore size, surface charges, intermolecular spaces in the network structure, and hydrophilic functional groups within the matrix. These factors determine how much aqueous solution the hydrogel can absorb and retain. Thus, the presence of OH and COOH groups from SA makes the network of SA/PA/PITAU hydrogels very water-friendly.

**Figure 3:**
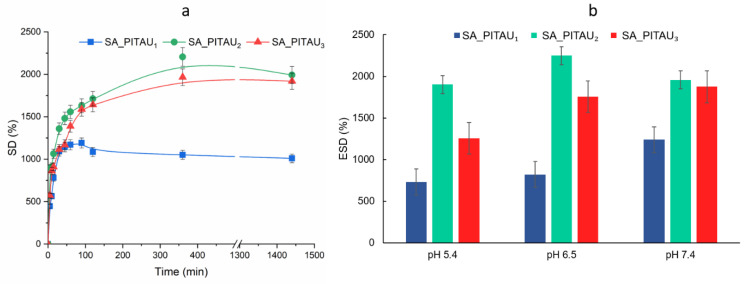
(**a**) Swelling kinetics of SA/PA/PITAU hydrogels in buffer solution with pH 7.4 at 25 °C; (**b**) effect of pH on the swelling behavior at equilibrium (25 °C).

The swelling behavior of SA/PA/PITAU hydrogels as a function of time and SA/PITAU ratio, in buffer solution with pH 7.4, at 25 °C, was studied and is shown in Figure 3a. As can be seen, all samples demonstrate a burst increase in swelling degree (SD) within the first 10 min caused by the osmotic pressure difference between the dried hydrogel and the solution. Subsequently, their SD continued to increase slowly for up to 24 h. Hydrogels swelling capacity increased proportionally with the SA content, except for the SA_PITAU_3_ sample, which showed a slight SD reduction. In the case of SA_PITAU_3_ hydrogel, reducing the PITAU ratio results in a decrease in swelling capacity compared to SA_PITAU_2_. This occurs due to the formation of additional crosslinks between the SA polymer segments and PA, which limits the available space for solvent molecules. This rigidity caused by the crosslinks restricts the relaxation of the chain, providing a potential explanation for the swelling behavior.

The sample with the lowest amount of SA (SA_PITAU_1_) and the highest amount of PITAU is the one with the lowest swelling capacity. The system’s compact architecture (also highlighted by the morphological characterization) caused the reduced swelling capacity.

The hydrogel with the intermediate quantity of PITAU and SA (SA_PITAU_2_) exhibited the highest SD. Compared to SA_PITAU_1_, a moderately smaller quantity of PITAU results in the formation of a more uniform network structure with interconnected pores that facilitate the diffusion of buffer molecules due to the relative mobility of the SA chains. Thus, the combination of adequate hydrophilicity provided by SA and the stabilizing effects of PA and PITAU resulted in a swelling capacity that can be suitable for wound care and drug delivery.

While the PA content reported to SA is maintained constant during hydrogel preparation, the increase in SA induces strong hydrophilicity through guluronic acid (G) units that provide ionizable moieties like OH and COOH, which can increase the volume between polymeric chains and the swelling capacity of the hydrogel by electrostatic repulsion.

To investigate the impact of pH on swelling behavior, hydrogel SD at equilibrium state was determined by using phosphate buffer solutions with different pHs corresponding to the wound healing stages (Figure 3b) [64].

As expected, the influence of pH media on SD at equilibrium depends on the ratio between SA and PITAU. All hydrogels had ESD increasing with the pH. SA has a pKa value of 3.6 and therefore the COOH groups over its pKa are ionized. Due to the conversion of the ionizable COOH groups of SA to a negatively charged COO^-^ ion and also to the resultant electrostatic forces, the ESD of hydrogels increases from pH 5.4 to pH 7.4. Moreover, in the studied pH domain, PA is highly negatively charged [65], which increases the density of intrinsic electrostatic repulsion forces.

In addition, a higher influx of counterions from swelling media at pH 7.4 can create a charge screening effect that also leads to a slight reduction in the charge repulsion effect and polyelectrolyte chain shrinkage (SA_PITAU_2_ and SA_PITAU_3_). This characteristic is valuable for bioactive compound (LVO) release in medical applications. The hydrogel behavior is in accordance with the SEM micrographs. Analogous swelling behaviors were observed for hydrogel dressings based on SA [66].

For LVO loading and further biological characterization, SA_PITAU_3_ was chosen due to a more controlled swelling capacity, which may favor a smaller burst release effect compared to SA_PITAU_2_, which exhibited greater water affinity.

### 3.4. Phytochemical Characterization of LVO from Lavender Flowers

LVO is known for its complex mixture of volatile organic compounds, and GC-MS is particularly well-suited to analyze such complex mixtures. Thus, GC-MS analysis of lavender oil revealed the following major compounds: linalool (17.71%), α-terpineol (7.31%), linalyl acetate (6.77%), 1,8-cineole (5.75%), 4-terpineol (4.89%), lavandulyl acetate (4.74%) (Table 3 and Figure S3).

### 3.5. LVD Loading/Release Studies

From the prepared hydrogels, SA_PITAU_3_ with an SA/PITAU ratio of 0.6:1 was chosen for LVO entrapment based on the swelling capacity and composition. EE% and LE% of different concentrations of LVO encapsulated in SA/PA/PITAU hydrogels are presented in Table 2. SA_PITAU_3_ hydrogel loaded with 10 wt% LVO revealed the highest EE% (88.11%). In addition, it was observed that EE% slightly decreased as LVO wt% in the matrix increased (87.06% for SA_PITAU_3_+LVO15%). Increasing the oil % in the hydrogels slightly decreased the EE% due to the reduced capacity of the polymer blend to sufficiently incorporate higher amounts of oil droplets. Thus, the excess of oil droplets remains on the surface of the hydrogels and is released quickly within the first hours. Other researchers have also reported that beyond a certain concentration, EE% of essential oils incorporation can begin to decline [67]. In addition, because the loading is carried out during the preparation of the hydrogel, there is a competition of interactions that occur between the active principles in the LVO and the components of the matrix, with those forming between SA polymer segments and PA groups that can hinder the LVO release process.

**Figure 4:**
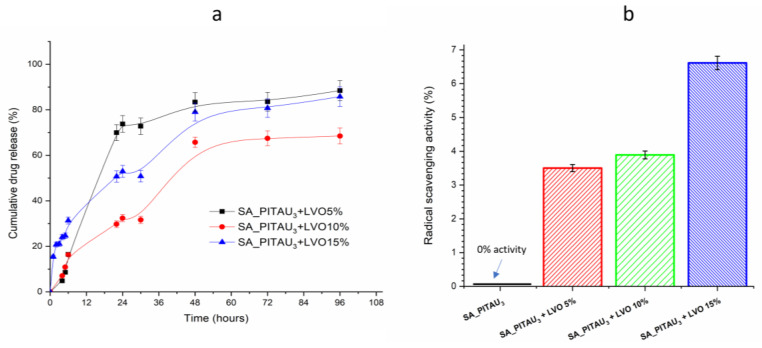
(**a**) Cumulative drug release curve of LVO-loaded hydrogels; (**b**) radical scavenging activity of LVO-loaded hydrogel and oil-free SA_PITAU_3_ hydrogel.

To mimic the internal biological environment, the in vitro drug release of LVO from hydrogels was studied in a buffer release medium (pH 7.4, 0.01 M). The release was monitored using UV-vis spectroscopy for 96 h at 276 nm under physiological conditions (Figure 4a).

First, as shown in Figure 4a, the LVO concentration in the buffer solution increases progressively over time, with an initial burst of loosely bounded oil particles to the SA/PITAU polymeric network released within 4 h. Further, a controlled release was observed up to 96 h, resulting in a cumulative release of 88.44%, 68.47%, and 86.77% from SA_PITAU_3_+LVO5%, SA_PITAU_3_+LVO10%, and SA_PITAU_3_+LVO15%, respectively. Comparing the results of the LVO release profile, SA_PITAU_3_+LVO5% is the one that releases the highest amount of LVO and is faster. Moreover, SA_PITAU_3_+LVO10% is the sample with the lowest LVO release in PBS after 96h, the last hydrogels also being the ones with the highest EE%. This is likely due to the fact that the remaining LVO oil entrapped in the core of the polymeric matrix releases slowly. Additionally, the hydrophobic nature of the oil constituents may also impede media from penetrating the deeper core, leading to a slower release. The release profile is in agreement with the findings of comparable studies conducted by other authors [68]. Thus, based on the cumulative release data, it can be concluded that LVO is released in two stages: an initial burst followed by a controlled diffusion. Given its antibacterial properties, it could be hypothesized that by releasing a large amount of LVO within the initial 24 h, bacterial infection significantly decreases. It may also lead to a reduction in the pain associated with injuries, as LVO has been reported to possess analgesic activity [63]. Later, a steady concentration of oil at the wound site aids in healing and providing antibacterial cover, as observed by other authors [68].

### 3.6. Antioxidant Activity

As reactive oxygen species (ROS) have a significant impact in modulating the wound healing response and the inflammatory processes, wound dressing materials with antioxidant properties, specifically those that scavenge radicals, are now considered essential [69]. Essential oils extracted from medicinal plants (like LVO) are rich in volatile compounds, including terpenoids, terpenes, aliphatic aromatic compounds, and phenolics, all of which contribute to their significant antioxidant capabilities. In Figure 4b, the radical scavenging activity reported to LVO concentration for loading the SA_PITAU_3_ hydrogel is represented.

The percentage of free radical scavenging activity of LVO-loaded hydrogels and pristine hybrid scaffold was measured by determining their potential to scavenge the DPPH (2,2-diphenyl-1-picrylhydrazyl) free radical by donating their electron or hydrogen atom and converting stable DPPH into its reduced form.

The results showed that all formulations containing LVO had antioxidant properties. Thus, the amount of essential oil employed to prepare the hydrogels has a direct correlation with the scavenging capacity. The highest antioxidant activity in hydrogels was obtained for SA_PITAU_3_+LVO15%, which was equal to 6.6%. The scavenging activity of LVO-loaded hydrogels was compared with the one of SA_PITAU_3_ hydrogel without essential oil, and the results showed that in the absence of LVO, the SA/PA/PITAU scaffold lacked antioxidant capacity. The drug release studies indicated a two-stage process with a burst effect in the first 5 h followed by a controlled diffusion that can also influence the scavenging capacity over time. The antioxidant assay results are very well correlated with the LC% data (Table 2) previously determined.

Prior investigations [70,71] reported higher LVO concentrations than were used in our experiments to produce suitable antioxidant activity. However, we discovered that despite using smaller amounts of LVO in SA/PA/PITAU, hybrid hydrogel still achieved good antioxidant activity.

Therefore, the SA/PA/PITAU polymeric hydrogels incorporated with LVO are an excellent option to scavenge the free radicals, which has the potential to significantly amplify the wound healing activity of hydrogels and sustain the anti-inflammatory process.

### 3.7. Antimicrobial Evaluation of Prepared Hybrid Hydrogels

**Figure 5:**
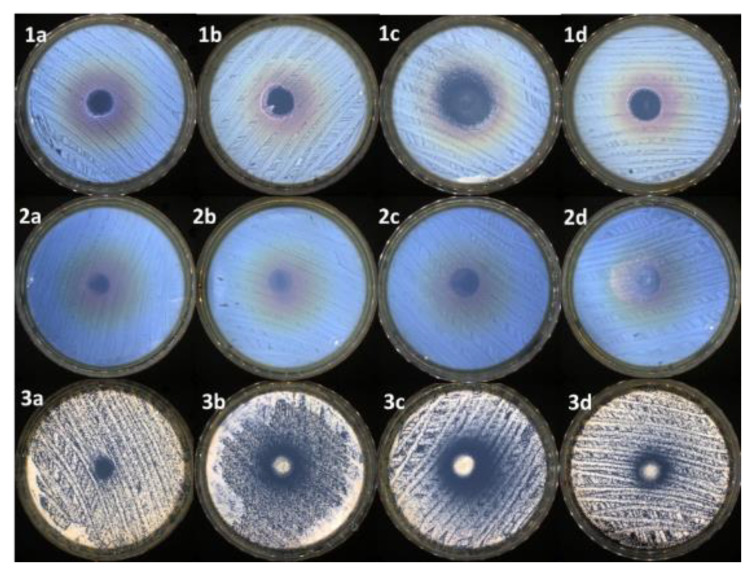
Antimicrobial activity of: (**1a**)—control against *S. aureus*; (**1b**)—sample SA_PITAU_3_+LVO5% against *S. aureus*; (**1c**)—sample SA_PITAU_3_+LVO10% against *S. aureus*; (**1d**)—sample SA_PITAU_3_+LVO15% against *S. aureus*; (**2a**)—control against *E. coli*; (**2b**)—sample SA_PITAU_3_+LVO5% against *E. coli*; (**2c**)—sample SA_PITAU_3_+LVO10% against *E. coli*; (**2d**)—sample SA_PITAU_3_+LVO15% against *E. coli*; (**3a**)—control against *C. albicans*; (**3b**)—sample SA_PITAU_3_+LVO5% against *C. albicans*; (**3c**)—sample SA_PITAU_3_+LVO10% against *C. albicans*; (**3d**)—sample SA_PITAU_3_+LVO15% against *C. albicans*.

As presented in Figure 5 and Table 4, almost all the samples presented antimicrobial activity against the tested reference strains. The smallest efficiency was presented by the control samples (up to 11 mm of inhibition zone). The addition of LVO improved the antimicrobial activity, up to a point, correlated with the oil concentration. Sample SA_PITAU_3_+LVO5% presented moderate antimicrobial activity (up to 15 mm of inhibition zone against *C. albicans*).

It was noticed that 10% of the oil in the case of SA_PITAU_3_+LVO10% sample led to the highest antimicrobial activity against the tested reference strains (up to 20 mm of inhibition zone in the case of *C. albicans*). Nevertheless, the highest concentration of LVO in the case of the SA_PITAU_3_+LVO15% sample determined a decrease in antimicrobial activity, to a level compared to SA_PITAU_3_+LVO5%. More than that, in the particular case of SA_PITAU_3_+LVO15 against *E. coli*, it can be observed that the sample determines only a small disturbance to the cells’ growth, without a proper inhibition zone.

The control’s antimicrobial activity (SA_PITAU_3_ sample without LVO) probably relies on the corroboration between SA and PA. PA is also known to have antimicrobial activity against several microbial species such as *E. faecalis*, *B. subtilis*, *S. aureus*, *P. aeruginosa*, *E. coli*, *S. mutans*, *S. typhimurium*, and *C. albicans* [72]. PA has antibacterial properties due to its cell membrane-damaging activity, which results from its surfactant properties provided by the content in phenolic compounds and flavonoids.

On the other hand, SA is also known to possess antimicrobial activity against bacterial strains such as *S. aureus* and *P. aeruginosa* [73]. Its antimicrobial mechanism can be attributed to the disruption of the intermolecular interaction in exopolysaccharides that might occur due to its negative charges [74].

LVO is presented in the literature as having remarkable antimicrobial activity against various microbial strains such as *S. aureus*, *B. cereus*, *P. aeruginosa*, *Candida* sp., *S. cerevisiae*, and *A. niger* [74]. The possible antimicrobial mechanism of the LVO includes damaging the cell wall and the membrane, and alteration of the proton motive force, leading in the end to the leakage of the cytoplasmic content [75]. LVO is also known to increase the synergistic effect of numerous classes of active agents [76].

Overall, very good antimicrobial activity was obtained in the case of the SA_PITAU_3_+LVO10% sample against *S. aureus* and *C. albicans* (see Figure 5 and Table 4). This efficiency is probably based on the synergism of SA, PA, and LVO. Therefore, due to its antimicrobial properties, the hydrogel SA_PITAU3+LVO10% was selected to be tested in terms of in vivo biocompatibility.

### 3.8. In Vivo Biocompatibility Testing

To assess the potential of the new hybrid hydrogels to be used as antibacterial wound dressings, their biocompatibility was evaluated in vivo on rats with subcutaneous implantation and the most relevant biochemical parameters of organs and tissue functions were monitored.

When tissues are damaged or infected with pathogenic germs, inflammation is rapidly initiated as an integrated immune process [77]. The main factors of this process are the complement system and innate phagocytes such as macrophages and dendritic cells. The activation of the complement system results in the assembly and deposition of membrane attack complexes that destroy infected or damaged cells by disrupting membrane integrity [78]. This process induces profound activation of phagocytes, with the production of inflammatory mediators such as prostaglandins, leukotrienes, and reactive oxygen species [79]. In addition, the production of bioactive complement fragments C3a and C5a, also known as anaphylatoxins, promotes local inflammatory events, including the recruitment of phagocytic cells to the site of inflammation [80]. These phagocytes work in conjunction with complement by binding and internalizing infectious agents through cell surface receptors and releasing proinflammatory cytokines (IL-1, TNF-α, and IL-6) [78].

**Figure 6:**
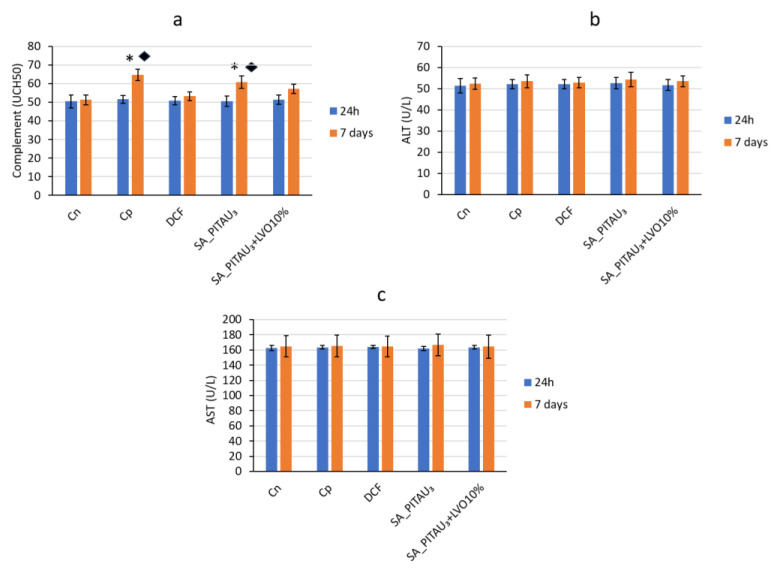
Influence of hydrogels on complement activity in the blood: (**a**) complemen; (**b**) ALT; (**c**) AST. * *p* < 0.05 versus time zero; ♦ *p* < 0.05 versus the control group.

At the beginning of the experiment, there were no variations regarding complement activity between the studied groups. Seven days after the induction of granuloma with cotton pellets, a significant increase in the serum level of complement was noted, in relation to the control group (Cn) (♦ *p* < 0.05) as well as to the initial moment (* *p* < 0.05). In the case of the group that received the SA_PITAU_3_ hydrogel, the activity of the serum complement increased substantially, after one week, compared to the control group without pellets (♦ *p* < 0.05), but also compared to the baseline (* *p* < 0.05) (Figure 6). There were no significant differences in blood complement levels between animals in the DCF-p group and no-pellet controls after one week.

After the application of the SA_PITAU_3_+LVO10% hydrogel, it was found that the plasma values of the complement increased modestly, without statistical relevance in relation to the control, and from time zero in the experiment (Figure 6a). This suggested that the immune system did not perceive the loaded hydrogel as foreign material. The influence of SA_PITAU3+LVO10% on complement activity was weaker than that determined by DCF-β over the course of the experiment.

ALT and AST activity did not differ significantly between SA_PITAU_3_, SA_PITAU_3_+LVO10%, pellet control, and no-pellet control at baseline and after one week (Figure 6b,c). This indicates that the normal liver function of the rats was not affected by the subcutaneous implantation of the hydrogels and therefore did not produce liver toxicity.

**Figure 7:**
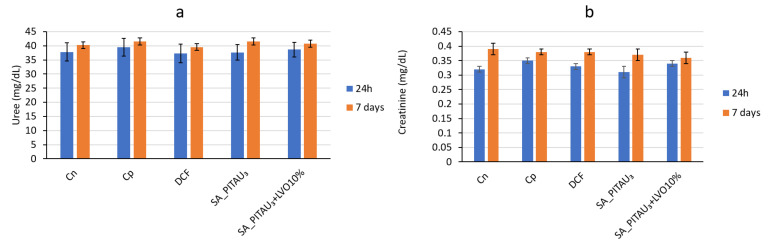
Influence of hydrogels on (**a**) blood urea and (**b**) creatinine levels.

There were no significant differences in blood urea and creatinine levels between the groups that received the studied hydrogels, the control group with granuloma, and the control group at the beginning of the experiment and after 7 days, indicating no kidney dysfunction (Figure 7a,b).

Before the start of the experiment, there were no substantial differences in blood MDA and SOD values between the SA_PITAU_3_ group, the SA_PITAU_3_+LVO10% group, the control group with pellets, and the control group without pellets.

**Figure 8:**
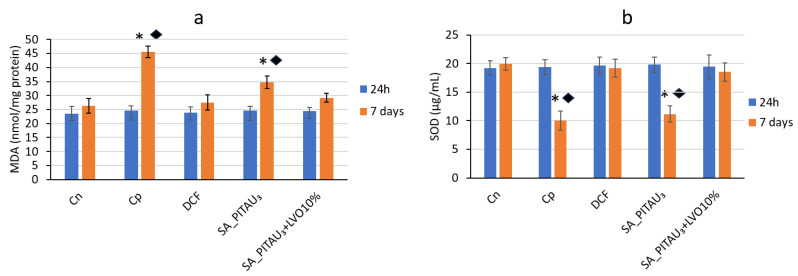
Influence of hydrogels on (**a**) MDA and (**b**) SOD activity. * *p* < 0.05 versus zero time; ♦ *p* < 0.05 versus the control group.

One week after the production of the granuloma, a significant increase (♦ *p* < 0.05) in the serum level of MDA and a significant reduction (* *p* < 0.05) in SOD values were observed, compared to the control group without granuloma, but also compared to the beginning of the experiment (* *p* < 0.05) (Figure 8).

Oxidative stress occurs when reactive oxygen species (ROS) are accumulated beyond the body’s antioxidant capacity. ROS can be produced as byproducts during normal metabolic processes, but in the case of inflammation, a significant accumulation of ROS may occur, causing an imbalance and the inability of cells to detoxify [81].

It is important to note that low levels of ROS are essential for maintaining critical biological processes, eliminating pathogenic microorganisms, and stimulating the growth of epithelial and fibroblastic cells. Increased concentrations of ROS create an environment of oxidative stress and promote a diversity of biological processes, such as neutrophil infiltration and fibroblast activation. Moreover, oxidative stress can also impact cell signaling pathways, which are essential for cell survival and repair [82].

ROS can interact with lipids to form MDA, which can modify proteins to form protein carbonyls. MDA is the most studied product of lipid peroxidation being considered a biomarker of oxidative stress.

The antioxidant system is widely present in human plasma and red blood cells and is divided into enzymes and non-enzymes. Key enzymes include glutathione reductase, peroxidase, catalase, glutathione S-transferase, SOD, glutathione peroxidase, tathion reductase, and thioredyne peroxidase. SOD forms the first line of defense against ROS-mediated damage [83]. These proteins catalyze the dismutation of superoxide anion free radicals into molecular oxygen and hydrogen peroxide and decrease the level of superoxide anion, which damages cells in excessive concentration. The enzyme acts as an important factor in preventing or ameliorating ROS-mediated diseases [84].

In the case of the DCF group, no suggestive changes in blood MDA and SOD levels were noted, compared to their without implant, after one week in the experiment.

In the case of the SA_PITAU_3_ hydrogel, after 7 days, the intensification in MDA activity and the decrease in SOD activity was highlighted with statistical relevance compared to the control (* *p* < 0.05) but also compared to the baseline (♦ *p* < 0.05) (Figure 8a,b).

The application of the SA_PITAU_3_+LVO10% hydrogel was accompanied by the improvement in disturbances in the activity of the two enzymes involved in lipid peroxidation after one week in the experiment.

The influence of SA_PITAU3+LVO10% on the serum values of MDA and SOD were less than those produced by DCF during the evaluation.

## 4. Conclusions

Hybrid hydrogels were prepared by mixing PITAU with SA that was crosslinked with PA. The physicochemical properties of the resulting hydrogels were investigated and the optimum hydrogel with the ratio of 0.6:1 between SA and PITAU was embedded with LVO to imprint antibacterial and antioxidant properties.

Through FTIR analysis, it was concluded that LVO was effectively incorporated into the hydrogels. The major characteristic band shifts confirmed the formation of hydrogen bonds between SA, the crosslinking agent (PA), and PITAU in the new hydrogels.

All freeze-dried hydrogels had a porous network with different morphology patterns, which were related to the variation in SA and PITAU ratio.

In comparison to SA_PITAU_1_, a moderate reduction in the quantity of PITAU results in the formation of a more uniform network structure with interconnected pores that facilitate the diffusion of buffer solution. Thus, the combination of adequate hydrophilicity provided by SA and the stabilizing effects of PA and PITAU resulted in a swelling capacity that is suitable for wound care and controlled drug delivery. The influence of pH media on SD at equilibrium depended on the ratio between SA and PITAU. All hydrogels were affected by the pH change in the swelling media, which impacted their ESD.

When comparing the results of the LVO release profile for the different hydrogels, it was found that SA_PITAU_3_+LVO5% released the highest amount of LVO and faster, while SA_PITAU_3_+LVO10% had the lowest LVO release in PBS after 96 h. The latter hydrogel also had the highest EE%. This is likely because the remaining LVO oil entrapped in the core of the polymeric matrix releases slowly and also because of the hydrophobic nature of LVO constituents, which may prevent release media from penetrating the deeper core.

The radical scavenging assay results showed that all formulations containing LVO had antioxidant properties. The highest antioxidant activity in hydrogels was obtained for SA_PITAU_3_+LVO15%.

The addition of LVO improved the antimicrobial activity, up to a point, correlated with the oil concentration. Very good antimicrobial activity was obtained in the case of the SA_PITAU_3_+LVO10% sample against *S. aureus* and *C. albicans.* The in vivo biocompatibility assay indicated that the application of the SA_PITAU_3_+LVO10% hydrogel had improved anti-inflammatory properties compared to oil-free SA_PITAU_3_ after one week in the experiment, but less intense than those of DCF, a frequently used anti-inflammatory drug.

In addition, the normal functions of the rats’ liver and kidney were not affected by the subcutaneous implantation of the hydrogels, so it did not produce liver toxicity or kidney dysfunction, confirming the biocompatibility of the new hydrogels loaded with LVO.

## Figures and Tables

**Table 1:** Codification and chemical composition of the studied hydrogels.

Sample Code	SA/PITAU (wt/wt)	SA/PA (wt/wt)
SA_PITAU_1_	0.3:1	6:1
SA_PITAU_2_	0.45:1	6:1
SA_PITAU_3_	0.6:1	6:1

**Table 2:** Codification and chemical composition of the SA_PITAU_3_ hydrogels loaded with LVO.

Sample Code	SA/PITAU (wt/wt)	LVO (wt%)	EE%	LC%	Radical Scavenging Capacity (%)
SA_PITAU_3_+LVO5%	0.6:1	5	83.74	33.33	3.5 ± 0.02
SA_PITAU_3_+LVO10%	0.6:1	10	88.11	50.44	3.9 ± 0.012
SA_PITAU_3_+LVO15%	0.6:1	15	87.06	58.11	6.6 ± 0.015

**Table 3:** LVO composition.

TR (min)	Compounds	Area %	TR (min)	Compounds	Area %
8.103	α-pinene	0.74	12.523	4-terpineol	4.89
8.436	Camphene	1.20	12.621	Crypton	0.52
8.965	β-pinene	0.60	12.774	α-terpineol	7.31
9.091	3-octanone	3.06	13.161	Nerol	2.59
9.162	β-myrcene	2.94	13.472	Linalyl acetate	6.77
9.549	Hexyl acetate	1.07	13.614	Geraniol	3.17
9.822	p-cymene	0.61	13.985	Lavandulyl acetate	4.74
9.909	Limonene	1.95	14.061	Bornyl acetate	0.64
9.980	1,8-Cineole	5.75	15.136	Neryl acetate	6.58
10.182	Trans-β-ocimene	2.23	16.162	β-caryophyllene	5.55
10.406	γ-terpinene	0.55	16.686	β-farnesene	0.91
10.635	Linalool oxide	1.69	17.101	germacren-D	1.11
10.875	Linalool	17.71	18.814	Caryophyllene oxide	1.57
11.999	Camphor	1.11	Other compounds (area < 0.5%)		8.36
12.184	Lavandulol	0.95			
12.408	Borneol	3.13			

**Table 4:** Antimicrobial activity of the tested compounds against the reference strains (mm).

Samples	Antimicrobial Activity (mm)		
	*S. aureus*	*E. coli*	*C. albicans*
Control (SA_PITAU_3_)	11.65 ± 0.21	9.00 ± 1.13	10.20 ± 0.42
SA_PITAU_3_+LVO5%	13.30 ± 1.69	12.60 ± 0.70	14.65 ± 1.62
SA_PITAU_3_+LVO10%	18.90 ± 1.69	15.10 ± 1.55	20.25 ± 2.47
SA_PITAU_3_+LVO15%	14.10 ± 2.26	-	11.00 ± 0.70

## Data Availability

Data are contained within the article.

## Supplementary Materials

The following supporting information can be downloaded at: https://www.mdpi.com/article/10.3390/pharmaceutics15112608/s1, Figure S1. Calibration curve of LVO loading, linearity range: 0.225–1.272 mg/mL. Figure S2. Calibration curve of LVO release, linearity range: 0.180–1.254 mg/mL. Figure S3. GC-MS chromatogram for LVO components from lavender flowers. Figure S4. The influence of hydrogels on the percentage values of leukocyte formula elements. * *p* < 0.05 vs. baseline, *♦ *p* < 0.05 vs. control group. Figure S5. Influence of hydrogels on blood fibrinogen and C-reactive protein values. Figure S6. Influence of hydrogels on serum levels of TNF-α and IL-10. * *p* < 0.05 versus time zero, ♦ *p* < 0.05 versus the control group. References [85,86,87] are cited in the supplementary materials.

## References

[1] Suflet D.M., Popescu I., Pelin I.M., David G., Serbezeanu D., Rîmbu C.M., Daraba O.M., Enache A.A., Bercea M. (2022). Phosphorylated Curdlan Gel/Polyvinyl Alcohol Electrospun Nanofibres Loaded with Clove Oil with Antibacterial Activity. Gels.

[2] Monfared-Hajishirkiaee R., Ehtesabi H., Rezaei A., Najafinobar S. (2023). Development of carboxymethyl cellulose/chitosan double-layer hydrogel combining myrtle essential oil and thyme honey to enhance antibacterial and mechanical properties. J. Ind. Eng. Chem..

[3] Chiriac A.P., Stoleru E., Rosca I., Serban A., Nita L.E., Rusu A.G., Ghilan A., Macsim A.-M., Mititelu-Tartau L. (2022). Development of a new polymer network system carrier of essential oils. Biomed. Pharmacother..

[4] Mostaghimi M., Majdinasab M., Golmakani M.-T., Hadian M., Hosseini S.M.H. (2023). Development and characterization of antimicrobial alginate hydrogel beads filled with cinnamon essential oil nanoemulsion. J. Biomater. Sci. Polym. Ed..

[5] Jia B., Li G., Cao E., Luo J., Zhao X., Huang H. (2023). Recent progress of antibacterial hydrogels in wound dressings. Mater. Today Bio.

[6] Herman A., Herman A.P. (2015). Essential oils and their constituents as skin penetration enhancer for transdermal drug delivery: A review. J. Pharm. Pharmacol..

[7] Garcia C.R., Malik M.H., Biswas S., Tam V.H., Rumbaugh K.P., Li W., Liu X. (2021). Nanoemulsion delivery systems for enhanced efficacy of antimicrobials and essential oils. Biomater. Sci..

[8] Yu S., Long Y., Li D., Shi A., Deng J., Ma Y., Wen J., Li X., Zhang Y., Liu S. (2022). Natural essential oils efficacious in internal organs fibrosis treatment: Mechanisms of action and application perspectives. Pharmacol. Res..

[9] Pinto E.P., Menezes R.P., Tavares W.d.S., Ferreira A.M., de Sousa F.F.O., da Silva G.A., Zamora R.R., Araújo R.S., de Souza T.M. (2023). Copaiba essential oil loaded-nanocapsules film as a potential candidate for treating skin disorders: Preparation, characterization, and antibacterial properties. Int. J. Pharm..

[10] Altay Ö., Köprüalan Ö., Ilter I., Koç M., Ertekin F.K., Jafari S.M. (2022). Spray drying encapsulation of essential oils; process efficiency, formulation strategies, and applications. Crit. Rev. Food Sci. Nutr..

[11] Koushik Y., Umashankar M.S. (2022). Nanoformulation Loaded with Essential Oils via Ultrasonication Technique: Overview, Challenges, and Prospects. Int. J. Pharm. Sci. Nanotechnol..

[12] Das S., Chaudhari A.K., Singh V.K., Dwivedy A.K., Dubey N.K. (2023). Chitosan based encapsulation of *Valeriana officinalis* essential oil as edible coating for inhibition of fungi and aflatoxin B1 contamination, nutritional quality improvement, and shelf life extension of *Citrus sinensis* fruits. Int. J. Biol. Macromol..

[13] Chiriac A.P., Rusu A.G., Nita L.E., Chiriac V.M., Neamtu I., Sandu A. (2021). Polymeric Carriers Designed for Encapsulation of Essential Oils with Biological Activity. Pharmaceutics.

[14] Hugar S.M., Gokhale N., Uppin C., Kajjari S., Meharwade P., Joshi R.S. (2022). The Effects of Lavender Essential Oil and its Clinical Implications in Dentistry: A Review. Int. J. Clin. Pediatr. Dent..

[15] Cavanagh H.M.A., Wilkinson J.M. (2002). Biological activities of Lavender essential oil. Phytother. Res..

[16] de Groot A., Schmidt E. (2016). Essential Oils, Part V: Peppermint Oil, Lavender Oil, and Lemongrass Oil. Dermatitis®.

[17] Hui L., He L., Huan L., Xiaolan L., Aiguo Z. (2010). Chemical composition of lavender essential oil and its antioxidant activity and inhibition against rhinitis-related bacteria. Afr. J. Microbiol. Res..

[18] de Rapper S., Viljoen A., van Vuuren S. (2016). The In Vitro Antimicrobial Effects of *Lavandula angustifolia* Essential Oil in Combination with Conventional Antimicrobial Agents. Evid.-Based Complement. Altern. Med..

[19] Jaramillo V., Díaz E., Muñoz L.N., González-Barrios A.F., Rodríguez-Cortina J., Cruz J.C., Muñoz-Camargo C. (2023). Enhancing Wound Healing: A Novel Topical Emulsion Combining CW49 Peptide and Lavender Essential Oil for Accelerated Regeneration and Antibacterial Protection. Pharmaceutics.

[20] Cavanagh H.M.A., Wilkinson J.M. (2005). Lavender essential oil: A review. Aust. Infect. Control.

[21] Government of India M.o.H. (1955). Pharmacopoeia of India: (The Indian Pharmacopoeia).

[22] Peana A.T., D’Aquila P.S., Panin F., Serra G., Pippia P., Moretti M.D.L. (2002). Anti-inflammatory activity of linalool and linalyl acetate constituents of essential oils. Phytomedicine.

[23] Tajik F., Eslahi N., Rashidi A., Rad M.M. (2021). Hybrid antibacterial hydrogels based on PVP and keratin incorporated with lavender extract. J. Polym. Res..

[24] Mori H.-M., Kawanami H., Kawahata H., Aoki M. (2016). Wound healing potential of lavender oil by acceleration of granulation and wound contraction through induction of TGF-β in a rat model. BMC Complement. Altern. Med..

[25] Ailincai D., Morariu S., Rosca I., Sandu A.I., Marin L. (2023). Drug delivery based on a supramolecular chemistry approach by using chitosan hydrogels. Int. J. Biol. Macromol..

[26] Iftime M.-M., Rosca I., Sandu A.-I., Marin L. (2022). Chitosan crosslinking with a vanillin isomer toward self-healing hydrogels with antifungal activity. Int. J. Biol. Macromol..

[27] Biddeci G., Cavallaro G., Di Blasi F., Lazzara G., Massaro M., Milioto S., Parisi F., Riela S., Spinelli G. (2016). Halloysite nanotubes loaded with peppermint essential oil as filler for functional biopolymer film. Carbohydr. Polym..

[28] Azzazy H.M.E.-S., Abdelnaser A., Al Mulla H., Sawy A.M., Shamma S.N., Elhusseiny M., Alwahibi S., Mahdy N.K., Fahmy S.A. (2023). Essential Oils Extracted from *Boswellia sacra* Oleo Gum Resin Loaded into PLGA–PCL Nanoparticles: Enhanced Cytotoxic and Apoptotic Effects against Breast Cancer Cells. ACS Omega.

[29] Diaconu A., Chiriac A.P., Nita L.E., Tudorachi N., Neamtu I., Vasile C., Pinteala M. (2015). Design and synthesis of a new polymer network containing pendant spiroacetal moieties. Des. Monomers Polym..

[30] Diaconu A., Rusu A., Nita L., Chiriac A., Neamtu I. (2017). Using riboflavin as low molecular mass gelator for the preparation of a new network structure having spiroacetal moieties. J. Res. Updat. Polym. Sci..

[31] Chiriac A.P., Nita L.E., Diaconu A., Neamtu I., Tudorachi N., Balan V. (2016). Matrix Copolymer Synthesis Process for Bio-Medical Applications.

[32] Farshidfar N., Iravani S., Varma R.S. (2023). Alginate-Based Biomaterials in Tissue Engineering and Regenerative Medicine. Mar. Drugs.

[33] Xie Y., Gao P., He F., Zhang C. (2022). Application of Alginate-Based Hydrogels in Hemostasis. Gels.

[34] Tavassoli-Kafrani E., Shekarchizadeh H., Masoudpour-Behabadi M. (2016). Development of edible films and coatings from alginates and carrageenans. Carbohydr. Polym..

[35] Kothale D., Verma U., Dewangan N., Jana P., Jain A., Jain D. (2020). Alginate as Promising Natural Polymer for Pharmaceutical, Food, and Biomedical Applications. Curr. Drug Deliv..

[36] Nair M.S., Tomar M., Punia S., Kukula-Koch W., Kumar M. (2020). Enhancing the functionality of chitosan- and alginate-based active edible coatings/films for the preservation of fruits and vegetables: A review. Int. J. Biol. Macromol..

[37] Malektaj H., Drozdov A.D., Christiansen J.D. (2023). Mechanical Properties of Alginate Hydrogels Cross-Linked with Multivalent Cations. Polymers.

[38] Kumar A., Dash G.K., Sahoo S.K., Lal M.K., Sahoo U., Sah R.P., Ngangkham U., Kumar S., Baig M.J., Sharma S. (2023). Phytic acid: A reservoir of phosphorus in seeds plays a dynamic role in plant and animal metabolism. Phytochem. Rev..

[39] Ghilan A., Nita L.E., Pamfil D., Simionescu N., Tudorachi N., Rusu D., Rusu A.G., Bercea M., Rosca I., Ciolacu D.E. (2022). One-Step Preparation of Carboxymethyl Cellulose—Phytic Acid Hydrogels with Potential for Biomedical Applications. Gels.

[40] Nita L.E., Chiriac A.P., Ghilan A., Rusu A.G., Tudorachi N., Timpu D. (2021). Alginate enriched with phytic acid for hydrogels preparation. Int. J. Biol. Macromol..

[41] Han D., Zhao H., Gao L., Qin Z., Ma J., Han Y., Jiao T. (2021). Preparation of carboxymethyl chitosan/phytic acid composite hydrogels for rapid dye adsorption in wastewater treatment. Colloids Surf. A Physicochem. Eng. Asp..

[42] Tashi Z., Zare M., Parvin N. (2020). Application of phytic-acid as an in-situ crosslinking agent in electrospun gelatin-based scaffolds for skin tissue engineering. Mater. Lett..

[43] Sandu A.E., Nita L.E., Chiriac A.P., Tudorachi N., Rusu A.G., Pamfil D. (2021). New Hydrogel Network Based on Alginate and a Spiroacetal Copolymer. Gels.

[44] Urbanova M., Pavelkova M., Czernek J., Kubova K., Vyslouzil J., Pechova A., Molinkova D., Vyslouzil J., Vetchy D., Brus J. (2019). Interaction Pathways and Structure–Chemical Transformations of Alginate Gels in Physiological Environments. Biomacromolecules.

[45] Davis T.A., Llanes F., Volesky B., Diaz-Pulido G., McCook L., Mucci A. (2003). 1H-NMR study of Na alginates extracted from Sargassum spp. in relation to metal biosorption. Appl. Biochem. Biotechnol..

[46] (2019). European Directorate for the Quality of Medicine and Health Care, Lavender Oil European Pharmacopoeia (Ph. Eur. 10.0).

[47] Yoon S.-W., Chung D.J., Kim J.-H. (2003). Preparation and swelling behavior of biodegradable hydrogels based on α,β-poly(*N*-2-hydroxyethyl-DL-aspartamide). J. Appl. Polym. Sci..

[48] Purcea Lopes P.M., Moldovan D., Moldovan M., Carpa R., Saroşi C., Păşcuţă P., Mazilu Moldovan A., Fechete R., Popescu V. (2022). New Composite Hydrogel Based on Whey and Gelatin Crosslinked with Copper Sulphate. Materials.

[49] Burhan A.M., Abdel-Hamid S.M., Soliman M.E., Sammour O.A. (2019). Optimisation of the microencapsulation of lavender oil by spray drying. J. Microencapsul..

[50] Mahmood H., Khan I.U., Khan R.U., Asghar S., Khalid I., Khalid S.H., Irfan M., Rehman F., Shahzad Y., Yousaf A.M. (2020). In vitro and in vivo evaluation of gellan gum hydrogel films: Assessing the co impact of therapeutic oils and ofloxacin on wound healing. Int. J. Biol. Macromol..

[51] Blois M.S. (1958). Antioxidant Determinations by the Use of a Stable Free Radical. Nature.

[52] Bauer A.W., Perry D.M., Kirby W.M.M. (1959). Single-disk antibiotic-sensitivity testing of staphylococci; an analysis of technique and results. A.M.A. Arch. Intern. Med..

[53] Clinical and Laboratory Standards Institute (CLSI) (2022). Performance Standards for Antimicrobial Susceptibility Testing.

[54] XLSTAT Statistical Software for Excel. https://www.xlstat.com/en.

[55] Carbone L., Bayne K., Turner P.V. (2014). Chapter 11-Euthanasia and Laboratory Animal Welfare. Laboratory Animal Welfare.

[56] Leary S.L., Underwood W.J., Anthony R., Cartner S.C., Corey D., Grandin T., Greenacre C.B., Gwaltney-Bran S., Mccrackin M.A., Meyer R.E. (2013). AVMA Guidelines for the Euthanasia of Animals.

[57] Zou W., Yang Y., Gu Y., Zhu P., Zhang M., Cheng Z., Liu X., Yu Y., Peng X. (2017). Repeated Blood Collection from Tail Vein of Non-Anesthetized Rats with a Vacuum Blood Collection System. J. Vis. Exp..

[58] Marques-Garcia F. (2020). Methods for Hemolysis Interference Study in Laboratory Medicine-A Critical Review. eJIFCC.

[59] Peskin A.V., Winterbourn C.C. (2017). Assay of superoxide dismutase activity in a plate assay using WST-1. Free Radic. Biol. Med..

[60] de Lima M.C., Marks G., Silva I.S., da Silva B.A.K., Cônsolo L.Z.Z., Nogueira G.B. (2012). Evaluation of oxidative stress in mice subjected to aerobic exercise. Acta Cir. Bras..

[61] Truzzi E., Durante C., Bertelli D., Catellani B., Pellacani S., Benvenuti S. (2022). Rapid Classification and Recognition Method of the Species and Chemotypes of Essential Oils by ATR-FTIR Spectroscopy Coupled with Chemometrics. Molecules.

[62] Sequeira R.S., Miguel S.P., Cabral C.S., Moreira A.F., Ferreira P., Correia I.J. (2019). Development of a poly(vinyl alcohol)/lysine electrospun membrane-based drug delivery system for improved skin regeneration. Int. J. Pharm..

[63] Sánchez E.C., García M.T., Pereira J., Oliveira F., Craveiro R., Paiva A., Gracia I., García-Vargas J.M., Duarte A.R.C. (2023). Alginate–Chitosan Membranes for the Encapsulation of Lavender Essential Oil and Development of Biomedical Applications Related to Wound Healing. Molecules.

[64] Norahan M.H., Pedroza-González S.C., Sánchez-Salazar M.G., Álvarez M.M., de Santiago G.T. (2023). Structural and biological engineering of 3D hydrogels for wound healing. Bioact. Mater..

[65] Nassar M., Nassar R., Maki H., Al-Yagoob A., Hachim M., Senok A., Williams D., Hiraishi N. (2021). Phytic Acid: Properties and Potential Applications in Dentistry. Front. Mater..

[66] Sun X., Ma C., Gong W., Ma Y., Ding Y., Liu L. (2020). Biological properties of sulfanilamide-loaded alginate hydrogel fibers based on ionic and chemical crosslinking for wound dressings. Int. J. Biol. Macromol..

[67] Yousefi M., Khanniri E., Sohrabvandi S., Khorshidian N., Mortazavian A.M. (2023). Encapsulation of Heracleum persicum essential oil in chitosan nanoparticles and its application in yogurt. Front. Nutr..

[68] Balasubramanian K., Kodam K.M. (2014). Encapsulation of therapeutic lavender oil in an electrolyte assisted polyacrylonitrile nanofibres for antibacterial applications. RSC Adv..

[69] Fadilah N.I.M., Phang S.J., Kamaruzaman N., Salleh A., Zawani M., Sanyal A., Maarof M., Fauzi M.B. (2023). Antioxidant Biomaterials in Cutaneous Wound Healing and Tissue Regeneration: A Critical Review. Antioxidants.

[70] Danh L.T., Han L.N., Triet N.D.A., Zhao J., Mammucari R., Foster N. (2013). Comparison of Chemical Composition, Antioxidant and Antimicrobial Activity of Lavender (*Lavandula angustifolia* L.) Essential Oils Extracted by Supercritical CO_2_, Hexane and Hydrodistillation. Food Bioprocess Technol..

[71] Marín I., Sayas-Barberá E., Viuda-Martos M., Navarro C., Sendra E. (2016). Chemical Composition, Antioxidant and Antimicrobial Activity of Essential Oils from Organic Fennel, Parsley, and Lavender from Spain. Foods.

[72] Nassar R., Nassar M., Vianna M.E., Naidoo N., Alqutami F., Kaklamanos E.G., Senok A., Williams D. (2021). Antimicrobial Activity of Phytic Acid: An Emerging Agent in Endodontics. Front. Cell. Infect. Microbiol..

[73] Asadpoor M., Ithakisiou G.-N., van Putten J.P.M., Pieters R.J., Folkerts G., Braber S. (2021). Antimicrobial Activities of Alginate and Chitosan Oligosaccharides against Staphylococcus aureus and Group B Streptococcus. Front. Microbiol..

[74] Nordgård C.T., Draget K.I. (2011). Oligosaccharides As Modulators of Rheology in Complex Mucous Systems. Biomacromolecules.

[75] Kavanaugh N.L., Ribbeck K. (2012). Selected Antimicrobial Essential Oils Eradicate Pseudomonas spp. and *Staphylococcus aureus* Biofilms. Appl. Environ. Microbiol..

[76] Arzi A., Ahamehe M., Sarahroodi S. (2011). Effect of hydroalcoholic extract of Lavandula officinalis on nicotine-induced convulsion in mice. Pak. J. Biol. Sci..

[77] Bardhan M., Kaushik R. (2023). Physiology, Complement Cascade. StatPearls.

[78] West E.E., Kemper C. (2023). Complosome—The intracellular complement system. Nat. Rev. Nephrol..

[79] Warwick C.A., Keyes A.L., Woodruff T.M., Usachev Y.M. (2021). The complement cascade in the regulation of neuroinflammation, nociceptive sensitization, and pain. J. Biol. Chem..

[80] Xu Z., Hou X.-F., Feng C.-M., Zheng L., Xu D.-X., Zhao H., Fu L. (2023). The association between serum complement C3a and severity in patients with community-acquired pneumonia. Front. Immunol..

[81] Chaudhary P., Janmeda P., Docea A.O., Yeskaliyeva B., Razis A.F.A., Modu B., Calina D., Sharifi-Rad J. (2023). Oxidative stress, free radicals and antioxidants: Potential crosstalk in the pathophysiology of human diseases. Front. Chem..

[82] Girón S.H., Sanz J.M., Ortega M.A., Garcia-Montero C., Fraile-Martínez O., Gómez-Lahoz A.M., Boaru D.L., de Leon-Oliva D., Guijarro L.G., Atienza-Perez M. (2023). Prognostic Value of Malondialdehyde (MDA) in the Temporal Progression of Chronic Spinal Cord Injury. J. Pers. Med..

[83] Husain S., Hillmann K., Hengst K., Englert H. (2023). Effects of a lifestyle intervention on the biomarkers of oxidative stress in non-communicable diseases: A systematic review. Front. Aging.

[84] Lu J., Guan S., Luo J., Yuan J., Yan J., Yang C., Tong Q. (2023). Levels of oxidative stress in patients with neoadjuvant chemotherapy for gastric cancer: Correlation with treatment response. Front. Oncol..

[85] van Loo G., Bertrand M.J.M. (2023). Death by TNF: A road to inflammation. Nat. Rev. Immunol..

[86] Montero-Blay A., Blanco J.D., Rodriguez-Arce I., Lastrucci C., Piñero-Lambea C., Lluch-Senar M., Serrano L. (2023). Bacterial expression of a designed single-chain IL-10 prevents severe lung inflammation. Mol. Syst. Biol..

[87] York A.G., Skadow M.H., Qu R., Oh J., Mowel W.K., Brewer J.R., Kaffe E., Williams K.J., Kluger Y., Crawford J.M. (2023). IL-10 constrains sphingolipid metabolism via fatty acid desaturation to limit inflammation. bioRxiv.

