# Supplementary material for: Alginate-Based Hydrogels Enriched with Lavender Essential Oil: Evaluation of Physicochemical Properties, Antimicrobial Activity, and In Vivo Biocompatibility
Source: Pharmaceutics. 2023 Nov 9;15(11):2608. doi: 10.3390/pharmaceutics15112608 (PMC10675056; doi:10.3390/pharmaceutics15112608)
Supplement: Supplementary file 1 [file pharmaceutics-15-02608-s001.zip › pharmaceutics-2662074-supplementary.pdf]

Supplementary materials

Alginate-Based Hydrogels Enriched with Lavender Essential Oil: Evaluation of Physicochemical Properties, Antimicrobial Activity, and In Vivo Biocompatibility

Alina Gabriela Rusu, Loredana Elena Niță, Irina Roșca, Alexandra Croitoriu, Alina Ghilan, Liliana Mititelu-Tarțău, Aurica Valentin Grigoraș, Bianca-Elena-Beatrice Crețu and Aurica P. Chiriac

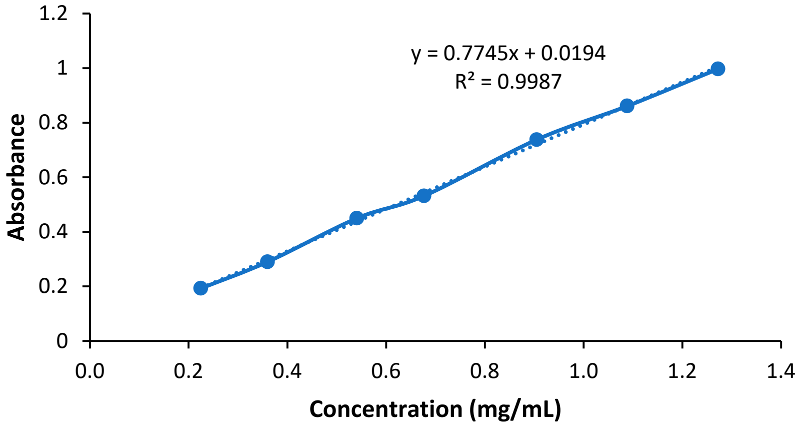

Figure S1. Calibration curve of LVO loading, linearity range: 0.225-1.272 mg/mL

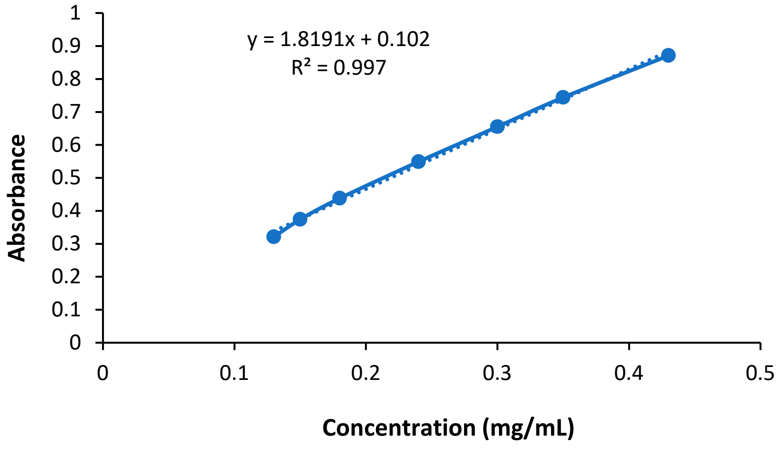

Figure S2. Calibration curve of LVO release, linearity range: 0.180-1.254 mg/mL

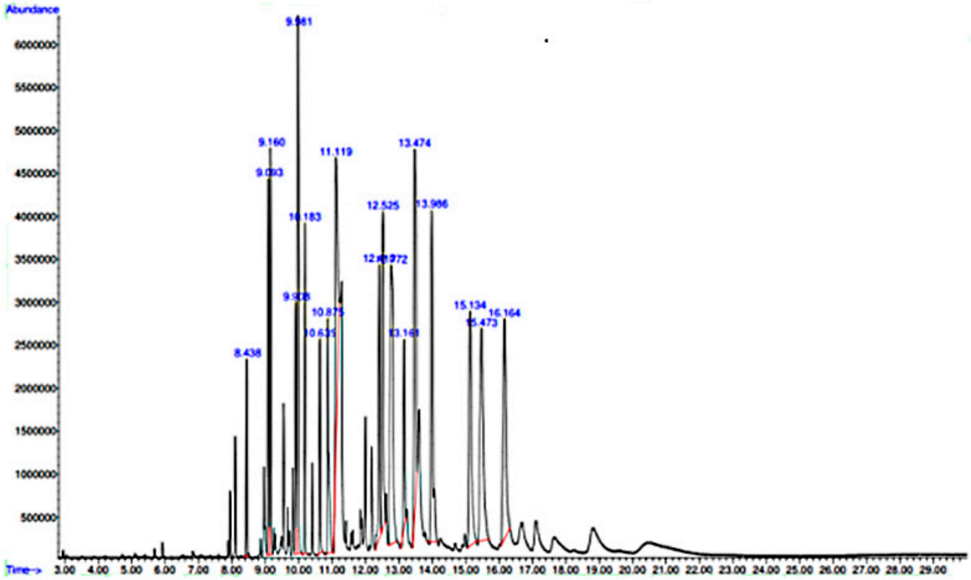

Figure S3. GC-MS chromatogram for LVO components from lavender flowers.

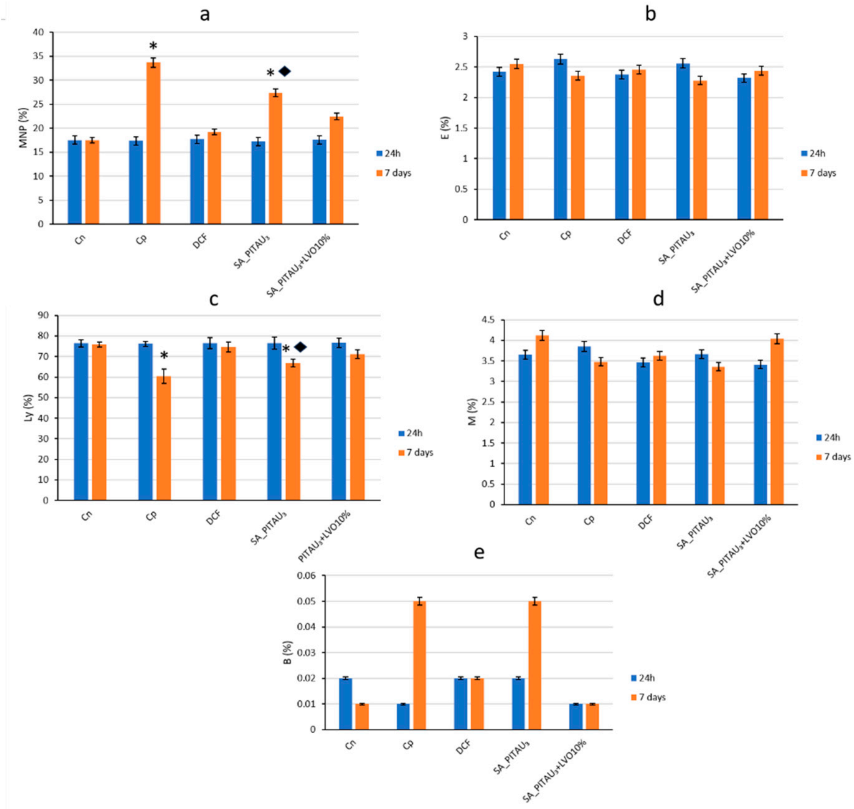

Figure S4. The influence of hydrogels on the percentage values of leukocyte formula elements: a) polymorphonuclear neutrophils (PMN), b) eosinophils (E), c) lymphocytes (Ly), d) monocytes (M), and e) basophils (B). *p<0.05 vs. baseline, *♦p<0.05 vs. control group.

No significant differences were detected between the percentage values of the elements of the leukocyte formula before the administration of the substances.
The use of SA_PITAU$_3$ hydrogel resulted in a significant increase in the percentage of PMN in the blood and at the same time a significant reduction in the percentage of Ly at 7 days, statistically significant compared to the control group (♦p<0.05), respectively compared to the initial moment (*p<0.05) in the experiment (Figure S4).
Subcutaneous application of SA_PITAU$_3$ +LVO10% hydrogel after one week produced a slight increase in the percentage of PMN and a modest decrease in the percentage of Ly, without statistical relevance compared to control and baseline, respectively.
Implantation of DCF-impregnated pellets was not associated with relevant changes in the percentage values of PMN and Ly, nor of other white cells, compared to the non-implanted group after one week.
Finally, after 7 days of the subcutaneous placement of SA_PITAU$_3$, respectively SA_PITAU$_3$ +LVO10% hydrogel, there were no obvious changes noted in the percentage values of E, M or B, compared to the control group without pellets, or compared to the initial moment (see Figure S4).
The influence of SA_PITAU$_3$ +LVO10% on the percentage values of PMN or Ly was less intense than that produced by DCF-β.
There were no significant differences in blood fibrinogen and C-reactive protein between groups. After Cp (cotton pellet) implantation, serum fibrinogen levels increased significantly. SA_PITAU$_3$ and SA_PITAU$_3$ +LVO10% hydrogel produced slight increases in plasma fibrinogen levels which were statistically insignificant. No significant differences in blood C-reactive protein values were found between the tested groups (Figure S5).

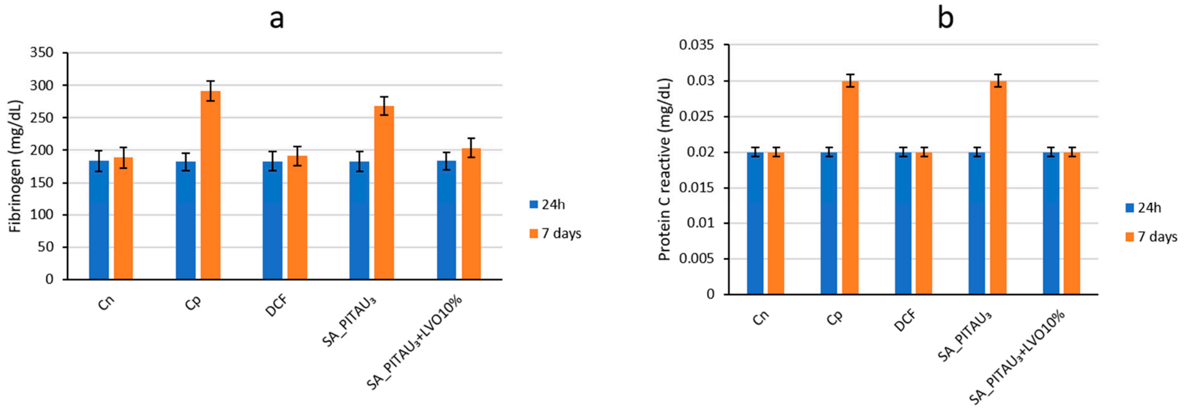

Figure S5. Influence of hydrogels on a) blood fibrinogen and b) C-reactive protein values.

There were no obvious variations at the beginning of the experiment regarding the plasma levels of TNF-α and IL-10 between the groups that received hydrogels and the control groups (Cn and Cp). However, after a week, the group that received cotton pellets exhibited a considerable increase (♦p<0.05) of TNF-α values and a decrease of IL-10 in the blood was highlighted, compared to the control group (Cn), respectively with the baseline (*p< 0.05) (Figure S6). The use of DCF-impregnated pellets did not result in significant changes in blood fibrinogen values compared to the control group without implant after one week in the experiment.

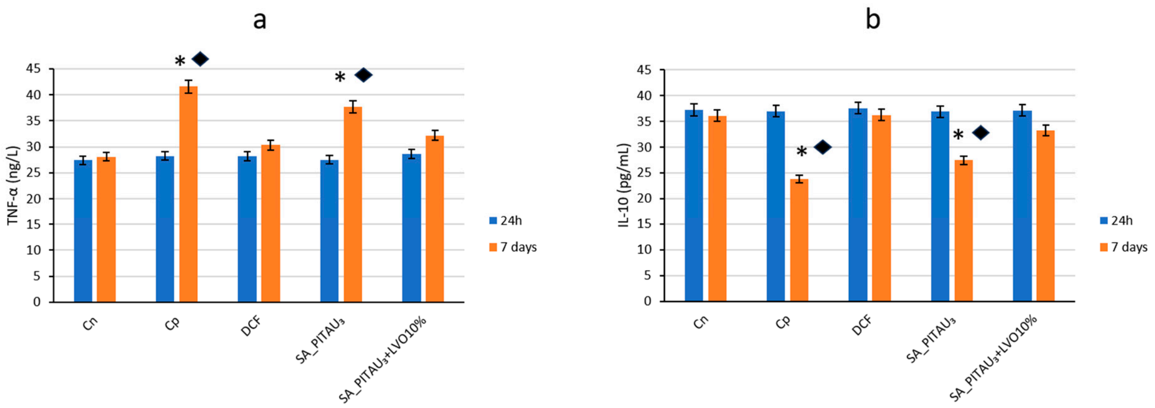

Figure S6. Influence of hydrogels on serum levels of a) tumor necrosis factor-alpha (TNFα) and b) interleukin 10 (IL-10). *$p<0.05$ versus time zero, ♦$p<0.05$ versus the control group.

TNF-α is a pro-inflammatory factor with an essential role in orchestrating the inflammatory immune response [1]. It is a multipotent cytokine produced by a wide range of immune cells such as B cells, T cells and macrophages. TNF-α is released immediately after injury, in case of infection or exposure to bacteria, being one of the most abundant early mediators in inflamed tissue [1]. IL-10 is an important immunoregulatory cytokine that acts to suppress inflammatory immune responses, largely by inhibiting the activation of monocytes and macrophages [2]. IL-10 plays an important role in attenuating inflammation or tissue damage by being produced by several white blood cell types, including lymphocytes, monocytes, and granulocytes, as well as non-immune cells such as epithelial or neuronal cells [3].

The use of SA_PITAU_3 hydrogel was associated after 7 days in the experiment with a considerable increase in the serum levels of TNF-α and a marked reduction in the levels of IL-10, compared to the control group with granuloma (♦$p<0.05$), respectively compared to the onset experiment (*$p<0.05$) (Figure 9).

Following subcutaneous application of SA_PITAU_3+LVO10% hydrogel, blood levels of TNF-α and IL-10 changed very little with no statistical significance compared to control and time zero.

Subcutaneous placement of DCF-impregnated pellets did not produce obvious changes in serum TNF-α and IL-10 compared to the non-implanted group after 7 days in the experiment.

1. van Loo, G.; Bertrand, M.J.M. Death by TNF: a road to inflammation. *Nature reviews. Immunology* **2023**, *23*, 289-303, doi:10.1038/s41577-022-00792-3.
2. Montero-Blay, A.; Blanco, J.D.; Rodriguez-Arce, I.; Lastrucci, C.; Piñero-Lambea, C.; Lluch-Senar, M.; Serrano, L. Bacterial expression of a designed single-chain IL-10 prevents severe lung inflammation. *Molecular systems biology* **2023**, *19*, e11037, doi:10.15252/msb.202211037.
3. York, A.G.; Skadow, M.H.; Qu, R.; Oh, J.; Mowel, W.K.; Brewer, J.R.; Kaffe, E.; Williams, K.J.; Kluger, Y.; Crawford, J.M.; et al. IL-10 constrains sphingolipid metabolism via fatty acid desaturation to limit inflammation. *bioRxiv : the preprint server for biology* **2023**, doi:10.1101/2023.05.07.539780.
